# Flight test results for microgravity active vibration isolation system on-board Chinese Space Station

**DOI:** 10.1038/s41526-024-00359-7

**Published:** 2024-02-19

**Authors:** Wei Liu, Yang Gao, Long Zhang, Tianji Zou, Mengxi Yu, Tuo Zheng

**Affiliations:** 1 Key Laboratory of Space Utilization, Technology and Engineering Center for Space Utilization, Chinese Academy of Sciences, Beijing, 100094 China; 2 University of Chinese Academy of Sciences, Beijing, 100049 China

**Keywords:** Aerospace engineering, Mathematics and computing, Statistical physics, thermodynamics and nonlinear dynamics, Techniques and instrumentation

## Abstract

The Fluid Physics Research Rack (FPR) is a research platform employed on-board the Chinese Space Station for conducting microgravity fluid physics experiments. The research platform includes the Microgravity Active Vibration Isolation System (MAVIS) for isolating the FPR from disturbances arising from the space station itself. The MAVIS is a structural platform consisting of a stator and floater that are monitored and controlled with non-contact electromagnetic actuators, high-precision accelerometers, and displacement transducers. The stator is fixed to the FPR, while the floater serves as a vibration isolation platform supporting payloads, and is connected with the stator only with umbilicals that mainly comprise power and data cables. The controller was designed with a correction for the umbilical stiffness to minimize the effect of the umbilicals on the vibration isolation performance of the MAVIS. In-orbit test results of the FPR demonstrate that the MAVIS was able to achieve a microgravity level of 1–30 μg_0_ (where g_0_ = 9.80665 m ∙ s^−2^) in the frequency range of 0.01–125 Hz under the microgravity mode, and disturbances with a frequency greater than 2 Hz are attenuated by more than 10-fold. Under the vibration excitation mode, the MAVIS generated a minimum vibration acceleration of 0.4091 μg_0_ at a frequency of 0.00995 Hz and a maximum acceleration of 6253 μg_0_ at a frequency of 9.999 Hz. Therefore, the MAVIS provides a highly stable environment for conducting microgravity experiments, and promotes the development of microgravity fluid physics.

## Introduction

The physical characteristics of fluids are dominated by gravity on the surface of the earth. As a result, the impacts of much smaller effects, such as capillarity, thermocapillarity, van der Waals forces, electrochemical/electrodynamic forces, Soret and Dufour effects, and contact line dynamics, are not readily observable. However, these unusual effects become dominant under the microgravity environments obtained in the low earth orbits of satellites and manned space stations. Moreover, microgravity environments provide a nearly ideal isotropic condition for conducting fluid research. As a result, the motion of fluids under microgravity conditions exhibits many characteristics and mechanisms not observable on the earth’s surface. For example, thermal convection on the surface of the earth, which is dominated by body force, differs considerably from that in space, which is dominated by surface force. This has led to increasing interest in research focusing on the motion characteristics of fluids and gases, such as heat and mass transfer processes, fluid dynamics, and complex fluid physics, observed under microgravity conditions and under variations in gravity^1,2^. Accordingly, numerous microgravity fluid physics experiments have been conducted on manned space stations^3^. The Influence of Vibrations on Diffusion in Liquids experiment on the International Space Station (ISS) was conducted to research the effect of random g-jitter and given vibrations on diffusion-controlled experiments in liquid mixtures. Experimental evidence disproved speculations that the ISS microgravity environment always affects diffusion-controlled processes, and an important conclusion that imposed vibrations with constant frequency and amplitude create slow mean flows and do influence the diffusion kinetics was demonstrated^4^. The acceleration levels required for various fluid physics experiments under microgravity conditions are summarized as a function of the corresponding vibrational acceleration levels^5^. Alternatively, microgravity acceleration levels are typically denoted as μg_0_, where g_0_ = 9.80665 m ∙ s^−2^. For example, the phase change and thermocapillary bubble migration have been conducted at around 1–100 μg_0_ and at acceleration frequencies on the order of 0.01–10 Hz. Similarly, the dynamics of hard spheres have been investigated at around 1000 μg_0_ and at acceleration frequencies on the order of 10–1000 Hz, while the rheology of non-Newtonian fluids have been require microgravity conditions on the order of 10,000 μg_0_.

In addition, special research platforms have been designed and installed in manned space stations for conducting microgravity fluid physics experiments^6,7^. Typically, these research platforms include vibration isolation systems as well to isolate the experimental platform from disturbances arising from the space station itself. Examples of these platforms and corresponding vibration isolation systems are listed in Table 1. As can be seen, the ISS includes a number of research platforms, including the Fluid Integrated Rack with its corresponding Active Rack Isolation System employed in the Destiny Laboratory Module of the US^8^, the Fluid Science Laboratory with its corresponding Microgravity Vibration Isolation Subsystem employed in the Columbus Laboratory of the European Space Agency^9^, and the RYUTAI Rack integrated with the Hope Experiment Module of the Japan Aerospace Exploration Agency. In addition, the Fluid Physics Research Rack (FPR), which is equipped with ten macroscale fluid dynamics test systems supporting a range of microgravity fluid physics experiments, is onboard the Mengtian laboratory cabin module of the Chinese Space Station (CSS) in conjunction with the Microgravity Active Vibration Isolation System (MAVIS). Microgravity experiments on the dynamic processes, diffusion processes, phase transitions, and self-organizing behaviors of different fluid systems will be conducted on MAVIS. In addition, MAVIS also supports interdisciplinary scientific and technological experimental research related to fluid thermal and mass transport in space material preparation and space biological processes. The MAVIS has six main operating modes, including a locked mode, central alignment mode, microgravity mode, vibration excitation mode, moving-to-locking-position mode, and fault mode.

**Table 1:** Experimental fluid physics devices installed on the International Space Station (ISS) and Chinese Space Station (CSS)

Fluid physics device	Research institute, country	Vibration isolation system	Spacecraft	Date of launch
Fluids Integrated Rack (FIR)	National Aeronautics and Space Administration (NASA), USA	Active Rack Isolation System (ARIS)	ISS	2009
Fluid Science Laboratory (FSL)	European Space Agency (ESA), EU	Microgravity Vibration Isolation Subsystem (MVIS)	ISS	2008
Ryutai Rack	Japan Aerospace Exploration Agency (JAXA), Japan	—	ISS	2011
Fluid Physics Research Pack (FPR)	China National Space Administration (CNSA), (China)	Microgravity Active Vibration Isolation System (MAVIS)	CSS	2022

The MAVIS employed in conjunction with the FPR is a structural platform consisting of a stator and a floater, which uses non-contact electromagnetic actuators, high-precision accelerometers, and displacement transducers for vibration isolation control. The stator is fixed to the FPR, while the floater serves as a vibration isolation platform supporting payloads, and is connected with the stator only with a number of umbilicals. However, the umbilicals, which mainly comprise power and data cables, have some stiffness, and inevitably provide pathways for the transfer of disturbance from the stator to the floater^10^. This represents a challenging condition for the MAVIS when operating in both its microgravity operating mode, which provides an environment with a controllable acceleration on the order of 1 μg_0_, and in its vibration excitation operating mode that provides an environment with controllable vibration acceleration signals of specific amplitudes in the frequency range of 0.01–10 Hz. These challenges were addressed by designing system controllers for the two operating modes of the MAVIS with a correction for the umbilical stiffness to minimize the effect of the umbilicals on the vibration isolation performance. The FPR was launched into orbit on October 31, 2022, installed on the CSS, and subjected to numerous tests to ascertain the performances of the designed control systems in both the microgravity and vibration excitation operating modes.

The present work presents the control system designs of the MAVIS in detail, and reports on the results of in-orbit testing. First, the hardware architecture of the MAVIS is described, and the requirements for its six operating modes and control performances are defined. Next, the control strategies for the microgravity and vibration excitation modes are explained in detail. Finally, the in-orbit test results of the MAVIS are summarized.

## Methods

### MAVIS components and requirements

**Fig. 1:**
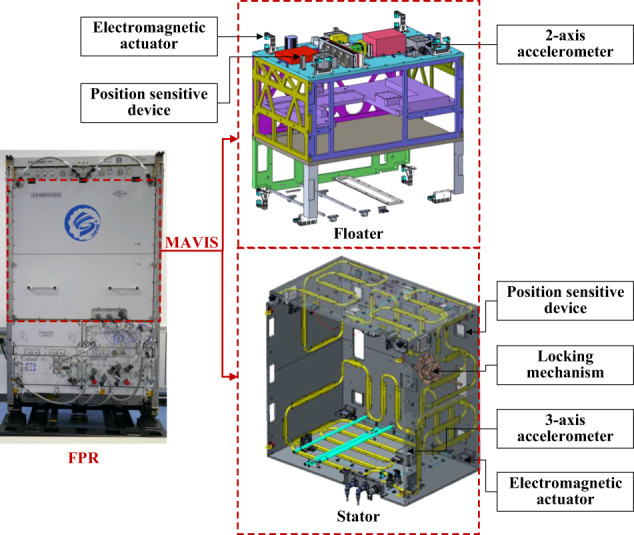
FPR and its MAVIS component installed in the Mengtian laboratory cabin module of the CSS.

The FPR and its MAVIS component currently installed in the Mengtian laboratory cabin module of the CSS is illustrated in Fig. 1, where the MAVIS is in its locked operating mode, which fixedly connects the floater and stator by a locking mechanism. The length × width × height dimensions of the MAVIS are approximately 600 mm × 950 mm × 940 mm. The floater and payload weigh approximately 132 kg.

The MAVIS senses vibrational accelerations on the experimental payload using accelerometers, and the motion of the floater/payload relative to the stator is measured using displacement transducers. Vibration isolation is achieved by transmitting the acceleration and relative motion information to the system controller, which uses a closed-loop control strategy to calculate the currents that must be applied to electromagnetic actuators to generate the appropriate opposing forces required to attenuate the magnitude of disturbances while avoiding collision between the floater and the stator. Four two-dimensional (2D) position sensitive devices (2D-PSDs) and eight one-dimensional (1D) electromagnetic actuators are configured between the stator and floater. In addition, electromagnetic levitation control is obtained by mounting three 2-axis accelerometers on the floater and one 3-axis accelerometer on the stator, as described in a previous report^11^. The displacement and attitude of the floater with respect to the stator measured by the 2D-PSDs is used for the single-loop displacement-based control (SDC), while the microgravity acceleration of the floater measured by the accelerometers and its displacement and attitude with respect to the stator measured by the 2D-PSDs are used for the two-loop impulse-averaging acceleration-based and displacement-based control (TIADC). Details regarding the measurement sensors and actuators employed in the control system are listed in Table 2, where the response frequency of the sensors refers to their measurement frequency, while that of actuators refers to their output frequency.

**Table 2:** Measurement sensors and actuators employed in the MAVIS

Component	Function	Response frequency	Precision
Accelerometer	Measuring microgravity acceleration	250 Hz	5 μg_0_ (0.01–125 Hz)
2D position sensitive device	Measuring the displacement and attitude of floater relative to that of stator	100 Hz	50 μm
1D electromagnetic actuator	Outputting control force and torque	250 Hz	Relative precision 10% in the force range of 0–5 mN and 1% in the force range of 5 mN–5 N

The functions and control strategies employed by the six operating modes of the MAVIS are summarized in Table 3, and are further described in detail as follows.

1. As discussed above, the floater and stator are fixedly connected by a locking mechanism under the locked mode. The MAVIS engages in no control operations in this mode, and the electromagnetic actuators are not activated. The locking mode is mainly used for protecting the floater and payloads during launching, rendezvous, and other operations affecting the CSS.
2. Under the central alignment mode, collision between the floater and stator is avoided by controlling the floater to remain fixed at the center of its available spatial range relative to the position of the stator. The available spatial range of the floater includes a vertical range of ±10 mm and rotational range of ±2°. This is the default mode at any microgravity level when the locking mechanism is released. In addition, the central alignment mode is activated when a preset safety threshold of relative displacement (e.g., 90%) is exceeded in the microgravity operating mode.
3. As discussed above, a high-level microgravity environment is created for experimental payloads in the microgravity operating mode through active vibration isolation control. The microgravity mode uses a two-loop impulse-averaging control strategy, where both the relative displacement/attitude and microgravity acceleration of the floater are subject to feedback control. This mode can also apply a single-loop strategy for controlling just the displacement of the floater.
4. As discussed above, the vibration excitation mode enables the application of vibrations of specific frequencies, magnitudes, and directions to experimental payloads. A single-loop control strategy based on displacement is applied for producing vibration signals in the frequency range of 0.01–1 Hz, while a two-loop control strategy based on both displacement and acceleration is applied for producing vibration signals in the frequency range of 0.1–10 Hz.
5. The moving-to-locking-position mode is activated when a test is terminated or as necessary under special circumstances. Under this mode, the floater is controlled to move rapidly to the designated locking position, and then connected to the stator by the locking mechanism. The moving-to-locking-position mode can be activated at any microgravity level, but the action time and overshoot of the control system are subject to specific requirements. The floater is set to move to the locking position within two minutes and without overshoot, which means there is no strong collision with the stator.
6. The fault mode is activated when a measurement transducer or electromagnetic actuator partially fails. Under this mode, the MAVIS either switches to a backup measurement system according to a failure response program or continues the closed-loop control according to the corresponding fault algorithm model. When the MAVIS experiences a severe failure, the floater is locked or the power is switched off via ground commands.

**Table 3:** Summary of the six operating modes of the MAVIS

Operating mode	Control strategy	Function
Locked mode	No control	Safety during launch and other operations
Central alignment mode	Single-loop displacement-based control (SDC)	Central alignment and collision avoidance after unlocking
Microgravity mode	Two-loop impulse-averaging acceleration-based and displacement-based control (TIADC), SDC	Provision of high-level microgravity environment
Vibration excitation mode	TIADC, SDC	Provision of specific vibrational acceleration signals
Moving-to-locking-position mode	SDC and trajectory planning	Moving floater to its locking position
Fault mode	Fault response plan, fault algorithm model	Fault identification and response

**Fig. 2:**
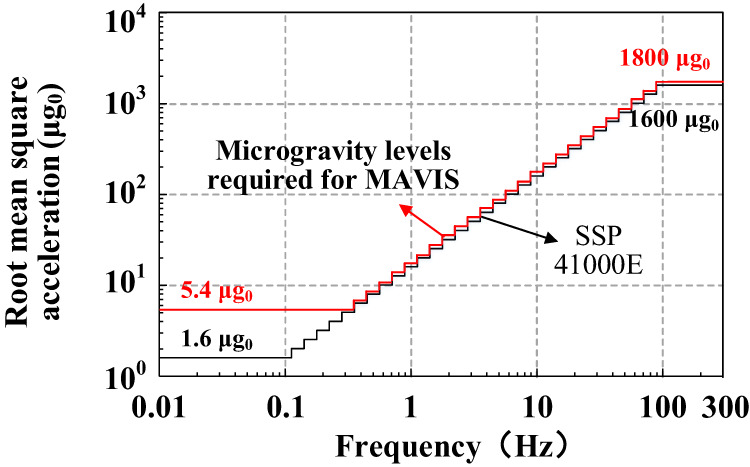
Microgravity acceleration requirements defined for payloads on the ISS in the SSP 41000E specification (black line) and for payloads subject to the MAVIS (red line) on the CSS.

The microgravity and vibration excitation modes are the primary operating modes of the MAVIS. The performance requirements applied for the microgravity mode follow from the microgravity acceleration requirements defined for payloads on the ISS in the SSP 41000E specification^12^. These requirements are summarized as follows. The microgravity acceleration at the center of the payload averaged over 100 s of operation in the microgravity operating mode must lie within the range of the root mean square (RMS) acceleration in the one-third octave indicated by the black line in Fig. 2. The microgravity acceleration performance required for the MAVIS is indicated by the red line in Fig. 2. The relationships between the microgravity acceleration *a* and vibration frequency *f* can be approximated as follows: (1) *a* ≤ 5.4 μg_0_ when 0.01 Hz ≤ *f* ≤ 0.3 Hz; (2) *a* ≤ 18 μg_0_ when 0.3 Hz < *f* ≤ 100 Hz; (3) *a* ≤ 1800 μg_0_ when 100 Hz < *f* ≤ 300 Hz.

The performance requirements applied for the vibration excitation mode include (1) a vibration frequency range of 0.01–10 Hz; (2) vibration types employing sine, triangular, and other classical periodic functions and time functions resulting from the superposition of two or more sine signals; (3) a maximum vibration acceleration ≥ 1500 μg_0_ in a given direction for a 100 kg payload.

### MAVIS dynamics models

The primary dynamics of the MAVIS were modeled using multiple coordinate systems, including the geocentric equatorial inertial system (***N***_*xyz*_), center of mass (CoM) orbit coordinate system (***O***_*xyz*_), spacecraft-fixed coordinate system (***B***_*xyz*_), stator-fixed coordinate system (***S***_*xyz*_), and floater-fixed coordinate system (***F***_*xyz*_). The following nonlinear model was established for defining the motion of the floater relative to the stator according to the Newton-Euler equations and the characteristics of rigid body composite motion.

1 $$\begin{array}{c}{{m}_{{\rm{F}}}\cdot {\ddot{{\boldsymbol{r}}}}_{{\rm{SF}}}|}_{S}+2{m}_{{\rm{F}}}\cdot {{\rm{N}}\atop}{\boldsymbol{\omega }}^{\rm{B}}\times {{\dot{{\boldsymbol{r}}}}_{{\rm{SF}}}|}_{S}+{m}_{{\rm{F}}}\cdot {{\rm{N}}\atop }{\boldsymbol{\alpha }}^{{\rm{B}}}\times {{\boldsymbol{r}}}_{{\rm{SF}}}\\ +{m}_{{\rm{F}}}\cdot {{\rm{N}}\atop }{\boldsymbol{\omega }}^{{\rm{B}}}\times ({{\rm{N}}\atop }{\boldsymbol{\omega }}^{{\rm{B}}}\times {{\boldsymbol{r}}}_{{\rm{SF}}})\\ ={{\boldsymbol{F}}}_{{\rm{Gra}}\_{\rm{F}}}-{{\boldsymbol{F}}}_{{\rm{Gra}}\_{\rm{S}}}+{{\boldsymbol{F}}}_{{\rm{Mag}}\_{\rm{F}}}+{{\boldsymbol{F}}}_{{\rm{Umb}}\_{\rm{F}}}+{{\boldsymbol{F}}}_{{\rm{Oth}}\_{\rm{F}}}-{{\boldsymbol{F}}}_{{\rm{Mic}}\_{\rm{S}}}\\ {{\boldsymbol{I}}}_{{\rm{F}}}\cdot ({{\rm{N}}\atop }{\boldsymbol{\alpha }}^{{\rm{B}}}+{{\rm{S}}\atop }{\boldsymbol{\alpha }}^{{\rm{F}}}+{{\rm{N}}\atop }{\boldsymbol{\omega }}^{{\rm{B}}}\times {{\rm{S}}\atop }{\boldsymbol{\omega }}^{{\rm{F}}})\\ +{({{\rm{N}}\atop }{\boldsymbol{\omega }}^{{\rm{B}}}+{{\rm{S}}\atop }{\boldsymbol{\omega }}^{{\rm{F}}})}^{\times }\cdot {{\boldsymbol{I}}}_{{\rm{F}}}\cdot ({{\rm{N}}\atop }{\boldsymbol{\omega }}^{{\rm{B}}}+{{\rm{S}}\atop }{\boldsymbol{\omega }}^{{\rm{F}}})\\ ={{\boldsymbol{M}}}_{{\rm{Gra}}\_{\rm{F}}}+{{\boldsymbol{M}}}_{{\rm{Mag}}\_{\rm{F}}}+{{\boldsymbol{M}}}_{{\rm{Umb}}\_{\rm{F}}}+{{\boldsymbol{M}}}_{{\rm{Oth}}\_{\rm{F}}}\end{array}$$

Here, *m*_F_ and ***I***_F_ are the mass and moment of inertia of the floater, respectively; ${{\boldsymbol{r}}}_{{\rm{SF}}}$ is the displacement of the floater relative to the stator, ${{\dot{{\boldsymbol{r}}}}_{{\rm{SF}}}|}_{S}$ and ${{\ddot{{\boldsymbol{r}}}}_{{\rm{SF}}}|}_{S}$ are the first-order and second-order differentials of ${{\boldsymbol{r}}}_{{\rm{SF}}}$ with respect to ***S***_*xyz*_, respectively; ${{\rm{S}}\atop}{\boldsymbol{\theta }}^{{\rm{F}}}$, ${{\rm{S}}\atop}{\boldsymbol{\omega }}^{{\rm{F}}}$, and ${{\rm{S}}\atop}{\boldsymbol{\alpha }}^{{\rm{F}}}$ are the attitude, body angular velocity, and attitude angular acceleration of the floater relative to the stator, respectively; ${{\rm{N}}\atop}{\boldsymbol{\omega }}^{{\rm{B}}}$, and ${{\rm{N}}\atop}{\boldsymbol{\alpha }}^{{\rm{B}}}$ are the body angular velocity and attitude angular acceleration of the CSS with respect to ***N***_*xyz*_, respectively; ${{\boldsymbol{F}}}_{{\rm{Gra}}\_{\rm{F}}}$, ${{\boldsymbol{F}}}_{{\rm{Mag}}\_{\rm{F}}}$, ${{\boldsymbol{F}}}_{{\rm{Umb}}\_{\rm{F}}}$, and ${{\boldsymbol{F}}}_{{\rm{Oth}}\_{\rm{F}}}$ are the forces acting on the floater due to celestial gravitation, the electromagnetic actuators, the umbilicals, and other disturbances, respectively; ${{\boldsymbol{F}}}_{{\rm{Gra}}\_{\rm{S}}}$ and ${{\boldsymbol{F}}}_{{\rm{Mic}}\_{\rm{S}}}$ are the celestial gravitation and non-conservative forces acting on the stator, respectively; ${{\boldsymbol{M}}}_{{\rm{Gra}}\_{\rm{F}}}$, ${{\boldsymbol{M}}}_{{\rm{Mag}}\_{\rm{F}}}$, ${{\boldsymbol{M}}}_{{\rm{Umb}}\_{\rm{F}}}$, and ${{\mathbf{M}}}_{{\rm{Oth}}\_{\rm{F}}}$ are the torques acting on the floater due to celestial gravitation, the electromagnetic actuators, the umbilicals, and other disturbances, respectively.

The disturbances from the umbilicals can be represented using the following second-order damping system^13^.

2 $$\begin{array}{l}{{\boldsymbol{F}}}_{{\rm{Umb}}\_{\rm{F}}}=\,\,-{{\boldsymbol{K}}}_{{\rm{tt}}}\cdot {{\boldsymbol{r}}}_{{\rm{SF}}}-{{\boldsymbol{C}}}_{{\rm{tt}}}{\cdot {\dot{{\boldsymbol{r}}}}_{{\rm{SF}}}|}_{{\rm{S}}}-{{\boldsymbol{K}}}_{{\rm{tr}}}\cdot {{\rm{S}}\atop}{\boldsymbol{\theta }}^{{\rm{F}}}\\\qquad\quad\quad\;\;\; -{{\boldsymbol{C}}}_{{\rm{tr}}}\cdot {{\rm{S}}\atop}{\boldsymbol{\omega }}^{{\rm{F}}}+{{\boldsymbol{F}}}_{{\rm{Umb}}\_{\rm{F}}0}\\ {{\boldsymbol{M}}}_{{\rm{Umb}}\_{\rm{F}}}=-{{\boldsymbol{K}}}_{{\rm{rt}}}\cdot {{\boldsymbol{r}}}_{{\rm{SF}}}-{{\boldsymbol{C}}}_{{\rm{rt}}}\cdot {{\dot{{\boldsymbol{r}}}}_{{\rm{SF}}}|}_{{\rm{S}}}-{{\boldsymbol{K}}}_{{\rm{rr}}}\cdot {{\rm{S}}\atop}{\boldsymbol{\theta }}^{{\rm{F}}}\\\qquad\quad\quad\;\;\;\, -{{\boldsymbol{C}}}_{{\rm{rr}}}\cdot {{\rm{S}}\atop}{\boldsymbol{\omega }}^{{\rm{F}}}+{{\boldsymbol{r}}}_{{\rm{FU}}}\times ({{\boldsymbol{F}}}_{{\rm{Umb}}\_{\rm{F}}}-{{\boldsymbol{F}}}_{{\rm{Umb}}\_{\rm{F}}0})\\\qquad\quad\quad\;\;\;\;+{{\boldsymbol{M}}}_{{\rm{Umb}}\_{\rm{F}}0}\end{array}$$

Here, ${{\boldsymbol{K}}}_{{\rm{tt}}}$, ${{\boldsymbol{K}}}_{{\rm{tr}}}$, ${{\boldsymbol{K}}}_{{\rm{rt}}}$, and ${{\boldsymbol{K}}}_{{\rm{rr}}}$ pertain to the stiffness matrices and ${{\boldsymbol{C}}}_{{\rm{tt}}}$, ${{\boldsymbol{C}}}_{{\rm{tr}}}$, ${{\boldsymbol{C}}}_{{\rm{rt}}}$, and ${{\boldsymbol{C}}}_{{\rm{rr}}}$ are the damping matrices of the umbilicals, ${{\boldsymbol{F}}}_{{\rm{Umb}}\_{\rm{F}}0}$ and ${{\boldsymbol{M}}}_{{\rm{Umb}}\_{\rm{F}}0}$ are the respective pretensioning force and torque of the umbilicals when the floater is located at the center of its available spatial range, and ${{\boldsymbol{r}}}_{{\rm{FU}}}$ is the position vector from the CoM of the floater to the equivalent point of action of the disturbance force of the umbilicals.

The nonlinear model was linearized to simplify the design and analysis of the controller. In addition, the translation equation was expanded in the ***S***_*xyz*_ coordinate system, and the rotation equation was expanded in the ***F***_*xyz*_ coordinate system. This yielded the following linear model.

3 $$\begin{array}{c}\left[\begin{array}{cc}{m}_{{\rm{F}}}\cdot {{\boldsymbol{E}}}_{3\times 3} & {{\boldsymbol{0}}}_{3\times 3}\\ {{\boldsymbol{0}}}_{3\times 3} & {({\rm{F}})\atop}{\boldsymbol{I}}_{{\rm{F}}}\end{array}\right]\left[\begin{array}{c}{{({\rm{S}})\atop}\ddot{{\boldsymbol{r}}}_{{\rm{SF}}}|}_{{\rm{S}}}\\ {({\rm{F}}){\rm{S}}\atop}{\boldsymbol{\alpha }}^{{\rm{F}}}\end{array}\right]\\ +\left[\begin{array}{cc}{{\boldsymbol{C}}}_{1}+{{\boldsymbol{C}}}_{{\rm{v}}} & {{\boldsymbol{C}}}_{2}\\ {{\boldsymbol{C}}}_{3} & {{\boldsymbol{C}}}_{4}+{{\boldsymbol{C}}}_{{\rm{\omega }}}\end{array}\right]\left[\begin{array}{c}{{({\rm{S}})\atop}\dot{{\boldsymbol{r}}}_{{\rm{SF}}}|}_{{\rm{S}}}\\ {({\rm{F}}){\rm{S}}\atop}{\boldsymbol{\omega }}^{{\rm{F}}}\end{array}\right]\\ +\left[\begin{array}{cc}{{\boldsymbol{K}}}_{1}+{{\boldsymbol{K}}}_{{\rm{x}}} & {{\boldsymbol{K}}}_{2}\\ {{\boldsymbol{K}}}_{3} & {{\boldsymbol{K}}}_{4}+{{\boldsymbol{K}}}_{{\rm{\theta }}}\end{array}\right]\left[\begin{array}{c}{({\rm{S}})\atop}{\boldsymbol{r}}_{{\rm{SF}}}\\ {({\rm{F}}){\rm{S}}\atop}{\boldsymbol{\theta }}^{{\rm{F}}}\end{array}\right]\\ =\left[\begin{array}{c}{({\rm{S}})\atop}{\boldsymbol{F}}_{{\rm{Mag}}\_{\rm{F}}}\\ {({\rm{F}})\atop}{\boldsymbol{M}}_{{\rm{Mag}}\_{\rm{F}}}\end{array}\right]+\left[\begin{array}{c}{{\boldsymbol{F}}}_{{\rm{a}}}\\ {{\boldsymbol{M}}}_{{\rm{\alpha }}}\end{array}\right]\,\end{array}$$

Here, ${({\rm{X}})\atop}{\boldsymbol{U}}$ designates the expansion of vector ${\boldsymbol{U}}$ in coordinate system X, with X designating the ***S***_*xyz*_ (S) or ***F***_*xyz*_ (F) coordinate system. Other parameters are defined in Table 4, where ${({\rm{O}})\atop}{\boldsymbol{\omega }}_{0}$ is the array of orbital angular velocities in the ***O***_*xyz*_ (O) coordinate system, $${{\boldsymbol{U}}}^{\times }=\left[\begin{array}{ccc}0 & -{U}_{3} & {U}_{2}\\ {U}_{3} & 0 & -{U}_{1}\\ -{U}_{2} & {U}_{1} & 0\end{array}\right]$$ is the antisymmetric matrix of vector ${\boldsymbol{U}}$, $${{\boldsymbol{W}}}_{0}=\left[\begin{array}{ccc}0 & 0 & 0\\ 0 & {\omega }_{0}^{2} & 0\\ 0 & 0 & -3{\omega }_{0}^{2}\end{array}\right]$$ is related to the magnitude of the orbital angular velocity ${\omega }_{0}$, and ${{\rm{Y}}\atop}{\boldsymbol{Q}}^{{\rm{X}}}$ is the coordinate transformation matrix from coordinate system X to coordinate system Y.

**Table 4:** Formulas of the parameters in the MAVIS linearization model

Parameter	Formula
***K***_1_	${({\rm{S}})\atop}{\boldsymbol{K}}_{{\rm{tt}}}$
***K***_2_	${({\rm{F}})\atop}{\boldsymbol{K}}_{{\rm{tr}}}$
***K***_3_	${({\rm{S}})\atop}{\boldsymbol{K}}_{{\rm{rt}}}+{({\rm{F}})\atop}{\boldsymbol{r}}_{{\rm{FU}}}^{\times }\cdot {({\rm{S}})\atop}{\boldsymbol{K}}_{{\rm{tt}}}$
***K***_4_	${({\rm{F}})\atop}{\boldsymbol{K}}_{{\rm{rr}}}+{({\rm{F}})\atop}{\boldsymbol{r}}_{{\rm{FU}}}^{\times }\cdot {({\rm{F}})\atop}{\boldsymbol{K}}_{{\rm{tr}}}$
***C***_1_	${({\rm{S}})\atop}{\boldsymbol{C}}_{{\rm{tt}}}$
***C***_2_	${({\rm{F}})\atop}{\boldsymbol{C}}_{{\rm{tr}}}$
***C***_3_	${({\rm{S}})\atop}{\boldsymbol{C}}_{{\rm{rt}}}+{({\rm{F}})\atop}{\boldsymbol{r}}_{{\rm{FU}}}^{\times }\cdot {({\rm{S}})\atop}{\boldsymbol{C}}_{{\rm{tt}}}$
***C***_4_	${({\rm{F}})\atop}{\boldsymbol{C}}_{{\rm{rr}}}+{({\rm{F}})\atop}{\boldsymbol{r}}_{{\rm{FU}}}^{\times }\cdot {({\rm{F}})\atop}{\boldsymbol{C}}_{{\rm{tr}}}$
***K***_x_	${m}_{{\rm{F}}}\cdot {{\rm{B}}\atop}{\boldsymbol{Q}}^{{\rm{O}}}\cdot {{\boldsymbol{W}}}_{0}\cdot {{\rm{O}}\atop}{\boldsymbol{Q}}^{{\rm{B}}}$
***K***_θ_	$$\begin{array}{c}-{[{({\rm{F}})\atop}{\boldsymbol{I}}_{{\rm{F}}}\cdot ({{\rm{B}}\atop}{\boldsymbol{Q}}^{{\rm{O}}}\cdot {({\rm{O}})\atop}{\boldsymbol{\omega }}_{0})]}^{\times }\cdot {({{\rm{B}}\atop}{\boldsymbol{Q}}^{{\rm{O}}}\cdot {({\rm{O}})\atop}{\boldsymbol{\omega }}_{0})}^{\times }\\ +{({{\rm{B}}\atop}{\boldsymbol{Q}}^{{\rm{O}}}\cdot {({\rm{O}})\atop}{\boldsymbol{\omega }}_{0})}^{\times }\cdot {({\rm{F}})\atop}{\boldsymbol{I}}_{{\rm{F}}}\cdot {({{\rm{B}}\atop}{\boldsymbol{Q}}^{{\rm{O}}}\cdot {({\rm{O}})\atop}{\boldsymbol{\omega }}_{0})}^{\times }\end{array}$$
***C***_v_	$2{m}_{{\rm{F}}}\cdot {{\rm{B}}\atop}{\boldsymbol{Q}}^{{\rm{O}}}\cdot {({\rm{O}})\atop}{\boldsymbol{\omega }}_{0}^{\times }\cdot {{\rm{O}}\atop}{\boldsymbol{Q}}^{{\rm{B}}}$
***C***_ω_	$$\begin{array}{c}{({\rm{F}})\atop}{\boldsymbol{I}}_{{\rm{F}}}\cdot {({{\rm{B}}\atop}{\boldsymbol{Q}}^{{\rm{O}}}\cdot {({\rm{O}})\atop}{\boldsymbol{\omega }}_{0})}^{\times }\\ +{({{\rm{B}}\atop}{\boldsymbol{Q}}^{{\rm{O}}}\cdot {({\rm{O}})\atop}{\boldsymbol{\omega }}_{0})}^{\times }\cdot {({\rm{F}})\atop}{\boldsymbol{I}}_{{\rm{F}}}\\ -{[{({\rm{F}})\atop}{\boldsymbol{I}}_{{\rm{F}}}\cdot {{\rm{B}}\atop}{\boldsymbol{Q}}^{{\rm{O}}}\cdot {({\rm{O}})\atop}{\boldsymbol{\omega }}_{0}]}^{\times }\end{array}$$
***F***_a_	$$\begin{array}{c}{({\rm{S}})\atop}{\boldsymbol{F}}_{{\rm{Umb}}\_{\rm{F}}0}+{({\rm{S}})\atop}{\boldsymbol{F}}_{{\rm{Oth}}\_{\rm{F}}}-{{\rm{B}}\atop}{\boldsymbol{Q}}^{{\rm{N}}}\cdot {{({\rm{N}})\atop}\ddot{{\boldsymbol{r}}}_{{\rm{NS}}}|}_{{\rm{N}}}\\ +{{\rm{B}}\atop}{\boldsymbol{Q}}^{O}\cdot {({\rm{O}})\atop}{\boldsymbol{\omega }}_{0}^{\times }\cdot {({\rm{O}})\atop}{\boldsymbol{\omega }}_{0}^{\times }\cdot {{\rm{O}}\atop}{\boldsymbol{Q}}^{{\rm{N}}}\cdot {({\rm{N}})\atop}{\boldsymbol{r}}_{{\rm{NS}}}\\ -{{\rm{B}}\atop}{\boldsymbol{Q}}^{{\rm{O}}}\cdot {{\boldsymbol{W}}}_{0}\cdot {{\rm{O}}\atop}{\boldsymbol{Q}}^{{\rm{B}}}\cdot {({\rm{B}})\atop}{\boldsymbol{r}}_{{\rm{BS}}}\end{array}$$
***M***_α_	$$\begin{array}{c}{({\rm{F}})\atop}{\boldsymbol{M}}_{{\rm{Gra}}\_{\rm{F}}}+{({\rm{F}})\atop}{\boldsymbol{M}}_{{\rm{Umb}}\_{\rm{F}}0}+{({\rm{F}})\atop}{\boldsymbol{M}}_{{\rm{Oth}}\_{\rm{F}}}\\ -{({{\rm{B}}\atop}{\boldsymbol{Q}}^{{\rm{O}}}\cdot {({\rm{O}})\atop}{\boldsymbol{\omega }}_{0})}^{\times }\cdot {({\rm{F}})\atop}{\boldsymbol{I}}_{{\rm{F}}}\cdot ({{\rm{B}}\atop}{\boldsymbol{Q}}^{{\rm{O}}}\cdot {({\rm{O}})\atop}{\boldsymbol{\omega }}_{0})\end{array}$$

### MAVIS control strategies

**Fig. 3:**
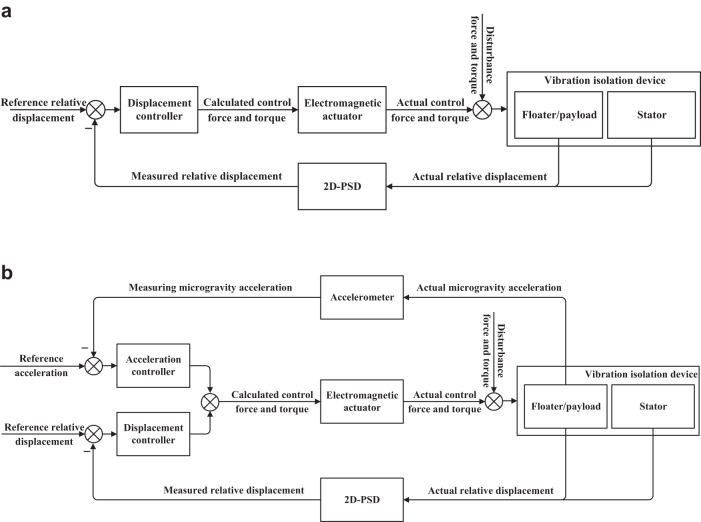
Block diagram of the two strategies employed by the MAVIS. **a** The SDC strategy. **b** The TIADC strategy.

As discussed above, the single-loop displacement-based control (SDC) and the two-loop impulse-averaging acceleration-based and displacement-based control (TIADC) strategies were employed to control the different operating modes of the MAVIS. The SDC strategy is illustrated in Fig. 3a, where the displacement and attitude of the floater with respect to the stator are controlled using a closed-loop feedback process. The TIADC strategy is illustrated in Fig. 3b, where the microgravity acceleration of the floater and its displacement and attitude with respect to the stator are controlled within a closed-loop feedback process.

The linearized dynamics model presented in Eq. (3) above was applied to establish the feedforward and feedback processes employed for the above-discussed single-loop and two-loop control strategies in terms of a proportional-integral-derivative (PID) controller. This yielded the following idealized control expressions.

4 $$\begin{array}{l}\left[\begin{array}{c}{({\rm{S}})\atop}{\boldsymbol{F}}_{{\rm{PC}}}\\ {({\rm{F}})\atop}{\boldsymbol{M}}_{{\rm{PC}}}\end{array}\right]={{\boldsymbol{K}}}_{{\rm{PC}}\_{\rm{P}}}\left[\begin{array}{c}{({\rm{S}})\atop}\varDelta {{\boldsymbol{r}}}_{{\rm{SF}}}\\ {({\rm{F}})\atop}\varDelta {{\rm{S}}\atop}{\boldsymbol{\theta }}^{{\rm{F}}}\end{array}\right]+{{\boldsymbol{K}}}_{{\rm{PC}}\_{\rm{D}}}\left[\begin{array}{c}{{({\rm{S}})\atop}\varDelta {\dot{{\boldsymbol{r}}}}_{{\rm{SF}}}|}_{{\rm{S}}}\\ {({\rm{F}})\atop}\varDelta {{\rm{S}}\atop}{\boldsymbol{\omega }}^{{\rm{F}}}\end{array}\right]\\ \quad\quad\quad+{{\boldsymbol{K}}}_{{\rm{PC}}\_{\rm{I}}}\left[\begin{array}{c}\int {({\rm{S}})\atop}\varDelta {{\boldsymbol{r}}}_{{\rm{SF}}}dt\\ \int {({\rm{F}})\atop}\varDelta {{\rm{S}}\atop}{\boldsymbol{\theta }}^{{\rm{F}}}dt\end{array}\right]-\left[\begin{array}{c}\,{({\rm{S}})\atop}{\boldsymbol{F}}_{{\rm{Umb}}\_{\rm{F}}0}\\ \,{({\rm{F}})\atop}{\boldsymbol{M}}_{{\rm{Umb}}\_{\rm{F}}0}\end{array}\right]\\ \quad\quad\quad+\left[\begin{array}{cc}{{\boldsymbol{K}}}_{1}+{{\boldsymbol{K}}}_{{\rm{x}}} \, {{\boldsymbol{K}}}_{2}\\ {{\boldsymbol{K}}}_{3} \, {{\boldsymbol{K}}}_{4}+{{\boldsymbol{K}}}_{{\rm{\theta }}}\end{array}\right]\left[\begin{array}{c}{({\rm{S}})\atop}{\boldsymbol{r}}_{{\rm{SF}}}\\ {({\rm{F}}){\rm{S}}\atop}{\boldsymbol{\theta }}^{{\rm{F}}}\end{array}\right]\\\quad\quad\quad+ \left[\begin{array}{cc}{{\boldsymbol{C}}}_{1}+{{\boldsymbol{C}}}_{{\rm{V}}} \, {{\boldsymbol{C}}}_{2}\\ {{\boldsymbol{C}}}_{3} \, {{\boldsymbol{C}}}_{4}+{{\boldsymbol{C}}}_{{\rm{\omega }}}\end{array}\right]\left.\right)\left[\begin{array}{c}{({\rm{S}})\atop}{\dot{\boldsymbol{r}}}_{{\mathrm{SF}}}|_{{\rm{S}}}\\ {({\rm{F}}){\rm{S}}\atop}{\boldsymbol{\omega }}^{{\rm{F}}}\end{array}\right]\\\quad\quad\quad+ \left[\begin{array}{c} {\rm{B}\atop}{\boldsymbol{Q}}^{\rm{O}} \cdot {\boldsymbol{W}}_{0}\cdot {\rm{O}\atop}{\boldsymbol{Q}}^{\rm{B}}\cdot {({\rm{B}})\atop}{\boldsymbol{r}}_{{\mathrm{BS}}}\\ ({\rm{B}\atop}{\boldsymbol{Q}}^{\rm{O}}\cdot {({\rm{O}})\atop}{\boldsymbol{\omega}}_{0})^{\times}\cdot {({\rm{F}})\atop}{\boldsymbol{I}}_{\rm{F}} \cdot ({{\rm{B}}\atop}{\boldsymbol{Q}}^{{\rm{O}}}\cdot {({\rm{O}})\atop}{\boldsymbol{\omega}}_{0}) \end{array}\right]\end{array}$$

5 $$\begin{array}{l}\left[\begin{array}{c}{({\rm{S}})\atop}{\boldsymbol{F}}_{{\rm{AC}}}\\ \,{({\rm{F}})\atop}{\boldsymbol{M}}_{{\rm{AC}}}\end{array}\right]={{\boldsymbol{K}}}_{{\rm{AC}}\_{\rm{Px}}}\left[\begin{array}{c}{({\rm{S}})\atop}\varDelta {{\boldsymbol{r}}}_{{\rm{SF}}}\\ {({\rm{F}})\atop}\varDelta {{\rm{S}}\atop}{\boldsymbol{\theta }}^{{\rm{F}}}\end{array}\right]+{{\boldsymbol{K}}}_{{\rm{AC}}\_{\rm{Dx}}}\left[\begin{array}{c}{{({\rm{S}})\atop}\varDelta {\dot{{\boldsymbol{r}}}}_{{\rm{SF}}}\Big|}_{{\rm{S}}}\\ {({\rm{F}})\atop}\varDelta {{\rm{S}}\atop}{\boldsymbol{\omega }}^{{\rm{F}}}\end{array}\right]\\ \qquad\qquad\qquad\quad+{{\boldsymbol{K}}}_{{\rm{AC}}\_{\rm{Ix}}}\left[\begin{array}{c}\int {({\rm{S}})\atop}\varDelta {{\boldsymbol{r}}}_{{\rm{SF}}}dt\\ \int {({\rm{F}})\atop}\varDelta {{\rm{S}}\atop}{\boldsymbol{\theta }}^{{\rm{F}}}dt\end{array}\right]-{{\boldsymbol{K}}}_{{\rm{AC}}\_{\rm{Pa}}}\left[\begin{array}{c}{({\rm{S}})\atop}\varDelta {{\boldsymbol{f}}}_{{\rm{Mic}}\_{\rm{F}}}\\ {({\rm{F}})\atop}\varDelta {{\rm{S}}\atop}{\boldsymbol{\alpha }}^{{\rm{F}}}\end{array}\right]\\ \qquad\qquad\qquad\quad-{{\boldsymbol{K}}}_{{\rm{AC}}\_{\rm{Ia}}}\left[\begin{array}{c}\int {({\rm{S}})\atop}\varDelta {{\boldsymbol{f}}}_{{\rm{Mic}}\_{\rm{F}}}dt\\ \int {({\rm{F}})\atop}\varDelta {{\rm{S}}\atop}{\boldsymbol{\alpha }}^{{\rm{F}}}dt\end{array}\right]-\left[\begin{array}{c}{({\rm{S}})\atop}{\boldsymbol{F}}_{{\rm{Umb}}\_{\rm{F}}0}\\ {({\rm{F}})\atop}{\boldsymbol{M}}_{{\rm{Umb}}\_{\rm{F}}0}\end{array}\right]\\ \qquad\qquad\qquad\quad+\left[\begin{array}{cc}{{\boldsymbol{K}}}_{1}+{{\boldsymbol{K}}}_{{\rm{x}}} \, {{\boldsymbol{K}}}_{2}\\ {{\boldsymbol{K}}}_{3} \, {{\boldsymbol{K}}}_{4}+{{\boldsymbol{K}}}_{{\rm{\theta }}}\end{array}\right]\left[\begin{array}{c}{({\rm{S}})\atop}{\boldsymbol{r}}_{{\rm{SF}}}\\ {({\rm{F}}){\rm{S}}\atop}{\boldsymbol{\theta }}^{{\rm{F}}}\end{array}\right]\\ \qquad\qquad\qquad\quad+\left[\begin{array}{cc}{{\boldsymbol{C}}}_{1}+{{\boldsymbol{C}}}_{{\rm{V}}} \, {{\boldsymbol{C}}}_{2}\\ {{\boldsymbol{C}}}_{3} \, {{\boldsymbol{C}}}_{4}+{{\boldsymbol{C}}}_{{\rm{\omega }}}\end{array}\right]\left.\right)\left[\begin{array}{c}{({\rm{S}})\atop}{\dot{\boldsymbol{r}}}_{{\mathrm{SF}}}|_{{\rm{S}}}\\ {({\rm{F}}){\rm{S}}\atop}{\boldsymbol{\omega }}^{{\rm{F}}}\end{array}\right]\\\qquad\qquad\qquad\quad +\left[\begin{array}{c} {\rm{B}\atop}{\boldsymbol{Q}}^{\rm{O}} \cdot {\boldsymbol{W}}_{0}\cdot {\rm{O}\atop}{\boldsymbol{Q}}^{\rm{B}}\cdot {({\rm{B}})\atop}{\boldsymbol{r}}_{{\mathrm{BS}}}\\ ({\rm{B}\atop}{\boldsymbol{Q}}^{\rm{O}}\cdot {({\rm{O}})\atop}{\boldsymbol{\omega}}_{0})^{\times}\cdot {({\rm{F}})\atop}{\boldsymbol{I}}_{\rm{F}} \cdot ({{\rm{B}}\atop}{\boldsymbol{Q}}^{{\rm{O}}}\cdot {({\rm{O}})\atop}{\boldsymbol{\omega}}_{0}) \end{array}\right]\end{array}$$

Here, ${{\boldsymbol{K}}}_{{\rm{PC}}\_{\rm{P}}}$, ${{\boldsymbol{K}}}_{{\rm{PC}}\_{\rm{D}}}$, and ${{\boldsymbol{K}}}_{{\rm{PC}}\_{\rm{I}}}$ are the PID controller parameters for the SDC strategy, and ${{\boldsymbol{K}}}_{{\rm{AC}}\_{\rm{Px}}}$,${{\boldsymbol{K}}}_{{\rm{AC}}\_{\rm{Dx}}}$, ${{\boldsymbol{K}}}_{{\rm{AC}}\_{\rm{Ix}}}$, ${{\boldsymbol{K}}}_{{\rm{AC}}\_{\rm{Pa}}}$, and ${{\boldsymbol{K}}}_{{\rm{AC}}\_{\rm{Ia}}}$ are the PID controller parameters for the TIADC strategy. However, the current design of the PID controller compensated only for the pretensioning force (${{\boldsymbol{F}}}_{{\rm{Umb}}\_{\rm{F}}0}$) and torque (${{\boldsymbol{M}}}_{{\rm{Umb}}\_{\rm{F}}0}$) of the umbilicals because the stiffness and damping matrices of the umbilicals are difficult to measure due to their flexibility, high nonlinearity, and hysteresis.

The PID controller parameter tuning problem was solved by first establishing the closed-loop transfer function from the stator’s microgravity acceleration to the floater’s microgravity acceleration, and then rewriting this function as a combination of some typical frequency elements, as proposed previously^14^. In the present work, the controller parameters were determined based on the control performance levels required for the different operating modes of the MAVIS. Accordingly, the PID controller parameters were calculated using the following equations.

6 $$\begin{array}{l}{{\boldsymbol{K}}}_{{\rm{PC}}\_{\rm{P}}}=({\omega }_{{\rm{n2}}}^{2}+2{\omega }_{{\rm{n1}}}{\omega }_{{\rm{n2}}}{\zeta }_{2})\times {{\boldsymbol{M}}}_{{\rm{diag}}}\\ {{\boldsymbol{K}}}_{{\rm{PC}}\_{\rm{I}}}\,={\omega }_{{\rm{n1}}}{\omega }_{{\rm{n2}}}^{2}\times {{\boldsymbol{M}}}_{{\rm{diag}}}\\ {{\boldsymbol{K}}}_{{\rm{PC}}\_{\rm{D}}}=({\omega }_{{\rm{n1}}}+2{\omega }_{{\rm{n2}}}{\zeta }_{2})\times {{\boldsymbol{M}}}_{{\rm{diag}}}\end{array}$$

7 $$\begin{array}{l}{{\boldsymbol{K}}}_{{\rm{AC}}\_{\rm{Pa}}}\;={A}_{{\rm{p}}}\times {{\boldsymbol{M}}}_{{\rm{diag}}}\\ {{\boldsymbol{K}}}_{{\rm{AC}}\_{\rm{Ia}}}\;\,=[({\omega }_{{\rm{n1}}}+2{\omega }_{{\rm{n2}}}{\zeta }_{2})-{\omega }_{{\rm{n1}}}{\omega }_{{\rm{n2}}}^{2}/{\omega }_{{\rm{n3}}}^{2}] \cdot (1+{A}_{{\rm{p}}})\times {{\boldsymbol{M}}}_{{\rm{diag}}}\\ {{\boldsymbol{K}}}_{{\rm{AC}}\_{\rm{Px}}}\,=({\omega }_{{\rm{n2}}}^{2}+2{\omega }_{{\rm{n1}}}{\omega }_{{\rm{n2}}}{\zeta }_{2})\cdot (1+{A}_{{\rm{p}}})\times {{\boldsymbol{M}}}_{{\rm{diag}}}\\ {{\boldsymbol{K}}}_{{\rm{AC}}\_{\rm{Ix}}}\;\,={\omega }_{{\rm{n1}}}{\omega }_{{\rm{n2}}}^{2}\cdot (1+{A}_{{\rm{p}}})\times {{\boldsymbol{M}}}_{{\rm{diag}}}\\ {{\boldsymbol{K}}}_{{\rm{AC}}\_{\rm{Dx}}}\,={\omega }_{{\rm{n1}}}{\omega }_{{\rm{n2}}}^{2}/{\omega }_{{\rm{n3}}}^{2}\cdot (1+{A}_{{\rm{p}}})\times {{\boldsymbol{M}}}_{{\rm{diag}}}\end{array}$$

Here, *ω*_n1_ is the natural frequency of the first-order inertia element; *ω*_n2_ and *ζ*_*2*_ are the natural frequency and damping ratio of the second-order oscillation element, respectively; ${{\boldsymbol{M}}}_{{\rm{diag}}}$ is 6-dimensional diagonal matrix, with the first three terms representing the mass of the floater and the last three terms representing the moment of inertia of the floater’s main axes; *A*_p_ is an adjustable parameter with a value in range of 0–1; *ω*_n3_ is the natural frequency of the second-order differentiation element. The vibration attenuation performance is determined by the parameters *ω*_n1_, *ω*_n2_, *ω*_n3_, and *ζ*_*2*_.

#### Microgravity mode

**Fig. 4:**
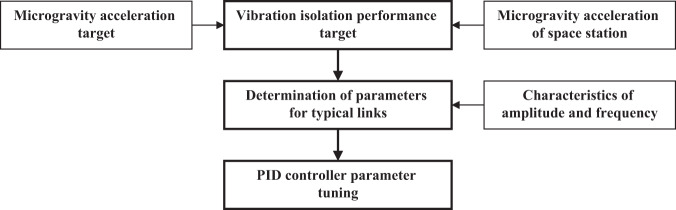
Flowchart of the process employed for tuning the PID controller parameters of the MAVIS.

The stiffness of the umbilicals determines the lower limit of the control system bandwidth. As discussed above, the control expressions of the PID controller are given in Eq. (5), and the PID controller parameters are calculated using Eq. (7). The effects of the parameters of the typical elements on the vibration isolation performance were then determined based on analysis of the amplitude-frequency characteristics, which are listed in Table 5. The vibrations at the frequencies less than the natural frequency *ω*_n1_ cannot be attenuated, and the vibration attenuation performance per ten octaves are approximately −20 dB above the natural frequency *ω*_n1_, −60 dB above the natural frequency *ω*_n2_, and −20 dB above the natural frequency *ω*_n3_. The PID controller parameters were then tuned according to the tuning process illustrated by the flowchart in Fig. 4. The procedure is given in detail as follows.

1. The microgravity acceleration target is set according to Fig. 2, and this target is combined with the estimated value for the actual microgravity acceleration of the space station^5^ to calculate the vibration isolation performance target, which is the attenuation of the microgravity acceleration target with respect to the microgravity acceleration of the space station. The vibration isolation performance target can be approximately described as follows: (a) for vibrations at frequencies less than 0.1 Hz, vibration attenuation is not required, and the maximum vibration magnification at the resonant frequency should not exceed 3 dB; (b) for vibrations in the frequency band of 0.1–10 Hz, the vibration attenuation is from 0 to −40 dB; (c) for vibrations at frequencies greater than 10 Hz, the vibration attenuation is −40 dB.
2. The vibration isolation performance target and the amplitude–frequency characteristics in Table 5 are combined to determine the parameters for typical elements. A set of preliminary design results that meet the requirements of the vibration isolation performance target are listed in Table 6.
3. The parameters obtained for typical elements are applied in Eq. (7) to calculate the PID controller parameters.

**Table 5:** Analysis of amplitude-frequency characteristics when evaluating the effects of PID controller parameters on the vibration isolation performance of the MAVIS based on the typical elements

Frequency	Amplitude
$\omega \,<\, {\omega }_{{\rm{n1}}}$	0 dB
${\omega }_{{\rm{n1}}}\le \omega \,<\, {\omega }_{{\rm{n2}}}$	−20 dB/dec
${\omega }_{{\rm{n2}}}\le \omega \,<\, {\omega }_{{\rm{n3}}}$	−60 dB/dec
$\omega \ge {\omega }_{{\rm{n3}}}$	−20 dB/dec

**Table 6:** Analysis of the impact of frequency, damping, and the adjustable parameter on the amplitude characteristics of the PID controller

Variable	Amplitude (rad/s)
*ω*_n1_	2π × 0.001
*ω*_n2_	2π × 0.05
*ω*_n3_	2π × 0.1
*ξ*_s_	0.8
*A*_p_	0.5

#### Vibration excitation mode

The amplitude-frequency characteristics of the SDC and TIADC strategies can be summarized as follows.

1. As discussed above, the SDC strategy is applied for generating vibrational acceleration signals in the relatively low frequency range of 0.01–1 Hz. This limitation is applied because the accuracy of the closed-loop control applied for providing the target displacement output is only sufficient at these relatively low frequencies.
2. Similar to (1) above, the TIADC strategy is applied for generating vibrational acceleration signals in the relatively high frequency range of 0.1–10 Hz owing the ability of this strategy to output acceleration signals accurately in this frequency range. The maximum amplitude of the floater’s vibration acceleration at a frequency *f* cannot exceed (2π*f*)^2^·*L*_lim_/2, where *L*_lim_ is the length of the spatial constraints.
3. For both control strategies, the amplitude of the actual output signal becomes attenuated relative to the desired amplitude of the target output when the frequency of the target output is close to the controller bandwidth. Therefore, the output signal is subjected to appropriate amplification to compensate for the impact of attenuation.

### Reporting summary

Further information on research design is available in the Nature Research Reporting Summary linked to this article.

## Results and discussion

After installing the FPR in the Mengtian laboratory cabin module in the CSS, the MAVIS was subjected to 13 days of in-orbit tests according to the procedures listed in Table 7. As can be seen, a full series of tests was performed using appropriate operation and control procedures, including self-check tests under the locked state, the testing of control algorithms, and the testing of microgravity and vibration excitation modes. The tests resulted in the tuning and optimization of 11 controller parameters, involved the input of approximately 2000 commands, and the output of approximately 15 GB of engineering data from the MAVIS.

**Table 7:** MAVIS in-orbit test procedures

Date	Test
2022.11.8	Self-check test under locked state
2023.1.9	Self-check test under locked state
2023.3.3	Self-check test under locked state
2023.3.24	SDC test, first trial of steady-state closed-loop control
2023.3.28	SDC test, optimization of controller parameters
2023.3.29	Optimization of controller parameters for SDC and TIADC
2023.4.10	Optimization of controller parameters for SDC and TIADC
2023.4.12	Test of microgravity and vibration excitation modes
2023.4.14	Test of microgravity and vibration excitation modes
2023.4.17–2023.4.20	Long-duration test of microgravity mode

### Self-check tests under the locked state

The three self-check tests under the locked state were conducted to evaluate the operational status of the MAVIS, including the heat sink, current, and voltage of the control board, and the functioning of the control components to ensure its readiness for closed-loop control after being unlocked. The control component functionality tests included the following.

**Fig. 5:**
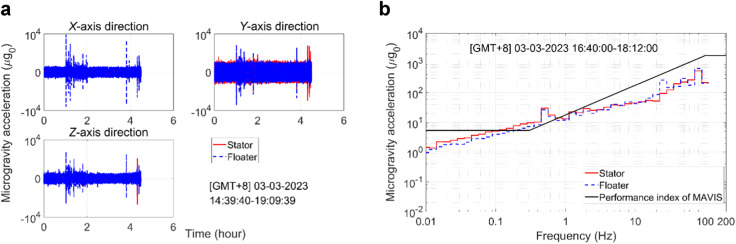
Accelerations measured by the accelerometers mounted on the floater and stator under the locked state during in-orbit tests. **a** Time series accelerations in the *X*, *Y*, and *Z* directions. **b** Root mean square (RMS) microgravity acceleration spectra.

1. Functioning of the four 2D-PSDs configured between the stator and floater and switching between the four measurement modes involving at least three 2D-PSDs applied for measuring the displacement and attitude of the floater relative to the stator. Here, measurement data is obtained from all four 2D-PSDs in the PSD41 measurement mode. Obtaining data from any three 2D-PSDs occurs under measurement modes denoted as PSD31, PSD32, and PSD33. Four measurement modes are used for mutual verification of accuracy and mutual backup. The PSD41 measurement mode is the default usage mode. If one 2D-PSD is faulty and the others are normal, the MAVIS will be switched to the measurement mode using the normal three 2D-PSDs. The in-orbit test results demonstrated that the displacements and attitudes of the floater relative to the stator measured using the four measurement modes were similar and highly consistent. The average displacement and attitude of the floater relative to the stator over the four measurement modes measured under the locked state in the *X*, *Y*, and *Z* directions were [−1.1 ± 0.1 mm 0.8 ± 0.05 mm −9.2 ± 1 mm]^T^ and [−0.15° ± 0.02° −0.06° ± 0.005° 0.51° ± 0.08°]^T^, respectively, and were consistent with the expected results. Accordingly, the 2D-PSDs were assumed to be functioning normally.
2. Functioning of the eight 1D electromagnetic actuators configured between the stator and floater. A 0.5 A control current input command was sent to each of the 1D electromagnetic actuators, and the actual currents of their energized coils were measured by current sensors. The in-orbit test results demonstrated that the measured currents were consistent with the control commands, which indicated that the electromagnetic actuators were functioning normally.
3. Functioning of the three 2-axis accelerometers on the floater and the one 3-axis accelerometer on the stator. The accelerations of the floater and stator must be equivalent under the locked state condition. Therefore, the accelerations measured by the accelerometers mounted on the floater were compared with the accelerations measured by the accelerometer mounted on the stator, and the results obtained in the *X*, *Y*, and *Z* directions during the in-orbit tests are presented in Fig. 5a. These results demonstrate that the measured accelerations of the floater were consistent with those of the stator. Accordingly, the accelerometers can be assumed to have been functioning normally. In addition, the RMS microgravity accelerations measured for the floater and stator under the locked state during the specified period of the in-orbit tests (i.e., 16:40–18:12 GMT + 8 [Beijing time]) are presented in Fig. 5b. As can be seen, the microgravity level of the floater under the locked state did not reach the level required for conducting microgravity experiments. Accordingly, active vibration isolation control was necessary.

### Testing of control algorithms

Under the microgravity mode, both control strategies use a small control bandwidth to minimize the disruption of the microgravity state of the floater by the control force. In contrast, both control strategies employed in the vibration excitation mode use a large control bandwidth to improve the accuracy of control. The data obtained from the control experiments conducted during the in-orbit tests can be analyzed as follows.

1. The time series variations of the control force and torque observed under displacement-based closed-loop control during the specified period of the in-orbit tests (i.e., 21:52–2:19 Beijing time) are presented in Fig. 6a, b, respectively. Accordingly, the values of the vectors ***F***_Umb_F0_ and ***M***_Umb_F0_ in the *X*, *Y*, and *Z* directions were [0.33 N − 0.26 N 0.16 N]^T^ and [0.026 N ∙ m 0.091 N ∙ m − 0.049 N ∙ m]^T^, respectively. As can be seen from Eq. (4), the values of ***F***_Umb_F0_ and ***M***_Umb_F0_ are approximately equal and opposite to the control force and torque under steady-state displacement-based closed-loop control. Accordingly, the floater is subject to disturbances from both the umbilicals and the control effects of the electromagnetic actuators during closed-loop control. Therefore, the natural frequency of the MAVIS is in effect determined by the stiffness of the umbilicals and the PID controller parameters. In fact, if the PID controller parameters are all set to zero, the natural frequency of MAVIS (equivalent to the control bandwidth) will be completely determined by the stiffness of the umbilical cables. Based on this analysis, we can conclude that the stiffness of the umbilicals has a significant impact on the control system bandwidth.
  The RMS microgravity accelerations measured for the floater and stator under the unlocked state, but when the position of the floater was not actively controlled, are presented in Fig. 6c for the specified period of the in-orbit tests (i.e., 9:15–10:47 Beijing time). As can be seen, the floater adheres to the surface of the stator under these conditions. This can be attributed to the disturbance effects of the umbilicals. In addition, the microgravity acceleration of the floater was markedly greater than that of the stator in the frequency range of 0.2–0.3 Hz. This frequency range corresponds to the natural frequency of the floater under the disturbance effects of the umbilicals, and therefore reflects the stiffness of the umbilicals.

**Fig. 6:**
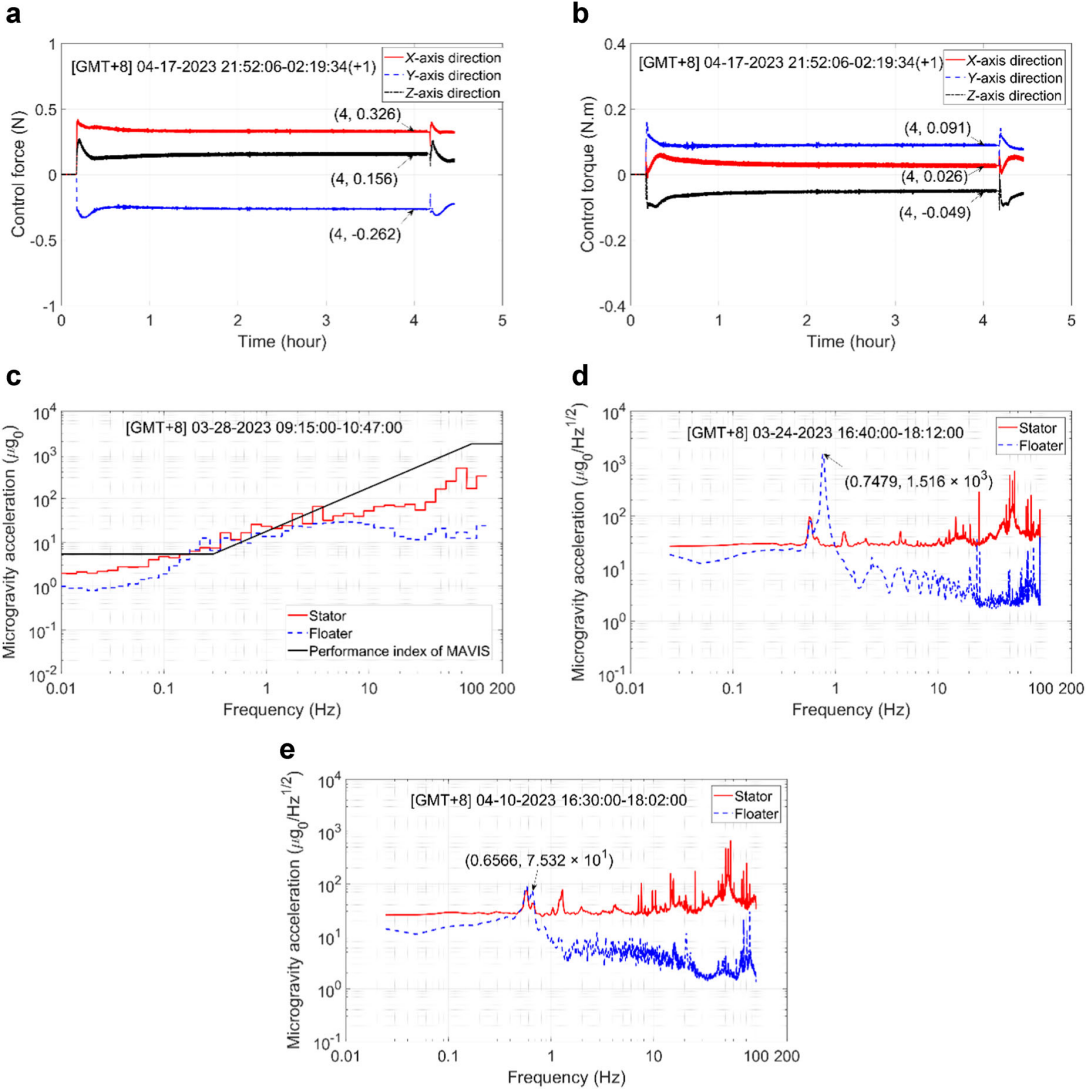
The data obtained from the control experiments conducted during the in-orbit tests for the analysis of the umblicals and attitude control. **a** Control force output under SDC. **b** Control torque output under SDC. **c** RMS microgravity acceleration spectra under the unlocked, uncontrolled state. **d** Power spectral density of the microgravity acceleration under relatively large controller parameters for attitude control. **e** Power spectral density of the microgravity acceleration under relatively small controller parameters for attitude control.
2. During closed-loop control of the MAVIS, both the translation and rotation of the floater are controlled based on the displacement and attitude of the stator. Moreover, the translational and rotational motions of the floater are coupled in the control process. In an effort to analyze the performance of closed-loop control, we first note from Eq. (2) that the disturbance force and torque of the umbilicals affect both the displacement and attitude of the floater relative to the stator. Second, floater control is conducted based on the force and torque output by the electromagnetic actuators. Therefore, a larger control torque can be expected to produce larger errors in the output torque and force. From this perspective, we investigated the natural frequency of the MAVIS at relatively large (*ω*_n2_ = 2π × 0.3) and small (*ω*_n2_ = 2π × 0.1) parameter values applied for attitude control in Fig. 6d, e, respectively, which was investigated in terms of the power spectral density of the microgravity acceleration observed for the floater and stator during in-orbit tests. As can be seen, the natural frequency of the MAVIS was approximately 0.75 Hz with a peak magnitude of about 1.516 × 10^−2^ under the relatively large control parameter values (Fig. 6d), while it was approximately 0.66 Hz with a peak magnitude of about 7.532 × 10^−4^ under the relatively small control parameter values (Fig. 6e). Therefore, the attitude control loop should be designed with relatively small control parameter values to promote the application of relatively small control torques that ensure maximum control accuracy.
3. Because of the large stiffness of the umbilicals, the parameter applied to the proportional term of the PID controller designed for the displacement control loop can be set to a suitable negative value, as long as the negative feedback of the umbilicals is greater than the positive feedback of the electromagnetic actuators. As a result, the MAVIS is subject to negative feedback control overall. Time series of the displacement and attitude of the floater measured in the *X*, *Y*, and *Z* directions with respect to (w.r.t.) that of the stator are presented in Fig. 7a, b, respectively, when applying a negative coefficient (Kp = −0.05*m*_F_) to the proportional term of the PID controller. The results demonstrate that the parameter applied for the proportional term achieved excellent closed-loop control stability that satisfied the constraints on the available spatial range of the floater ( ± 10 mm and ±2°). The RMS microgravity acceleration spectra measured for the floater using different parameter values (Kp) for the proportional term of the PID controller are presented in Fig. 7c. The results confirm that a negative value of Kp improved the microgravity level of the floater.

**Fig. 7:**
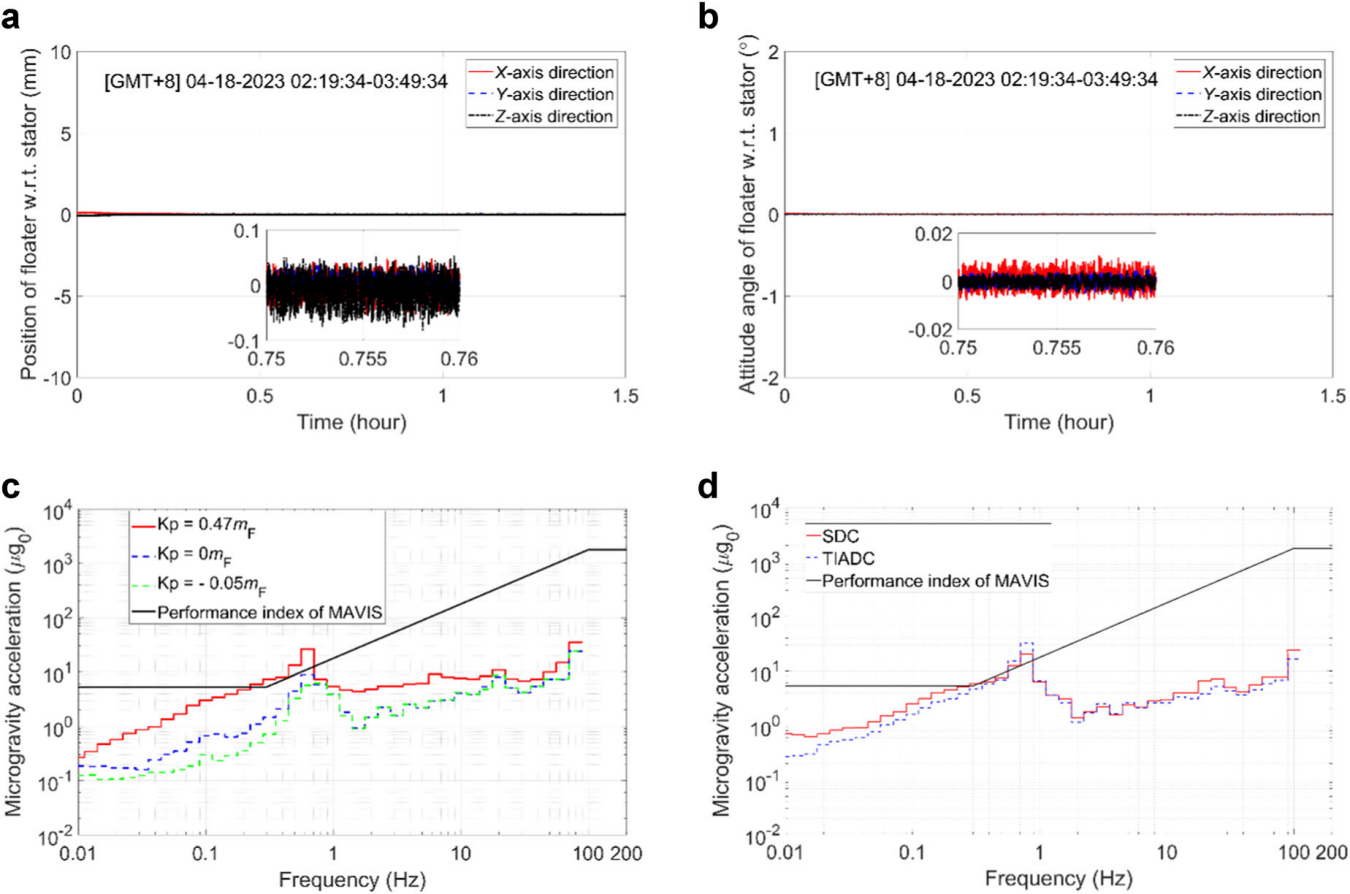
The data obtained from the control experiments conducted during the in-orbit tests for the analysis of the controller parameters and control strategies. **a** Displacement of the floater with respect to (w.r.t.) that of the stator when applying a negative coefficient to the proportional term of the PID controller. **b** Attitude of the floater w.r.t. that of the stator when applying a negative coefficient to the proportional term of the PID controller. **c** RMS microgravity acceleration spectra measured for the floater using different parameter values for the proportional term of the PID controller. **d** RMS microgravity acceleration spectra measured for the floater under different control strategies.
4. The RMS microgravity acceleration spectra measured for the floater under the SDC and TIADC strategies during in-orbit testing are presented in Fig. 7d. Here, the same PID controller parameter values were applied for the displacement controllers in both control strategies. As can be seen, the TIADC strategy outperformed the SDC strategy in terms of vibration isolation over the entire frequency range of 0.01–100 Hz, except for near the natural frequency of the floater near the range 0.6–0.9 Hz. Accordingly, the in-orbit test results confirm the effectiveness of the TIADC strategy in improving the vibration isolation of the floater.

### Tests of major operating modes

#### Microgravity mode

**Fig. 8:**
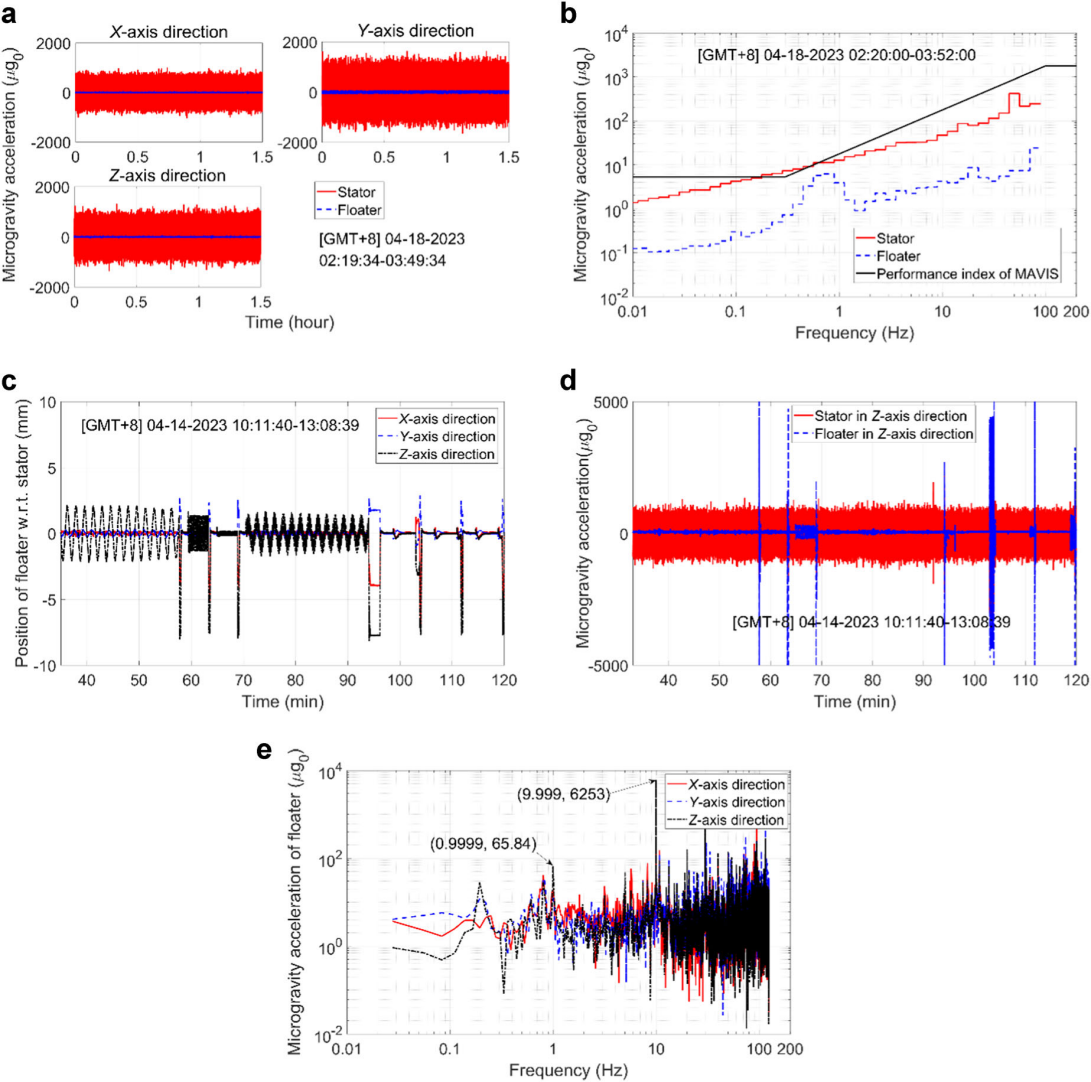
Flight test results under the microgravity mode and the vibration excitation mode. **a** Time series of the microgravity accelerations under the microgravity mode. **b** RMS microgravity acceleration spectra under the microgravity mode. **c** Time series of the displacement of the floater w.r.t. that of the stator under the vibration excitation mode. **d** Time series of the microgravity accelerations of the floater and stator under the vibration excitation mode. **e** Fourier transform of the microgravity accelerations of the floater under the vibration excitation mode.

The time series of the microgravity accelerations measured for the floater and stator in the *X*, *Y*, and *Z* directions and their corresponding RMS microgravity acceleration spectra observed under the microgravity mode are presented in Fig. 8a, b, respectively. The excellent vibration isolation performance of the MAVIS is clearly demonstrated by the significantly smaller microgravity accelerations of the floater than those of the stator (Fig. 8a). Meanwhile, the RMS microgravity acceleration spectrum of the floater (Fig. 8b) exhibits a microgravity acceleration of 0.1–30 μg_0_ in the frequency range of 0.01–125 Hz, which is markedly greater than that required for many microgravity experiments^5^.

#### Vibration excitation mode

The in-orbit test results obtained for the MAVIS under the vibration excitation mode are listed in Table 8 for sine waves, triangular waves, and mixed sine waves at frequencies ranging from 0.01 to 10 Hz and with different amplitudes and displacements. In particular, the MAVIS achieved a minimum microgravity amplitude of 0.41 μg_0_ at a frequency of 0.01 Hz, and maximum microgravity amplitudes of 4355 μg_0_ at a frequency of 9.6 Hz and 6253 μg_0_ at a frequency of 10 Hz when mixed with a sine wave at a frequency of 1.0 Hz. Time series of the displacement of the floater relative to that of the stator obtained during in-orbit testing under the vibration excitation mode are presented in Fig. 8c. Meanwhile, time series of the microgravity accelerations of the floater and stator obtained in the *Z* direction under the vibration excitation mode during in-orbit testing are presented in Fig. 8d. These results demonstrate the stability and effectiveness of the two control strategies in this operation mode. In addition, a Fourier transform of the microgravity accelerations of the floater measured in the *X*, *Y*, and *Z* directions under the vibration excitation mode is shown in Fig. 8e. As can be seen, the frequency response of the microgravity accelerations achieve maxima at 0.9999 Hz and 9.999 Hz, which demonstrates that the MAVIS can generates vibration signals at specified frequencies.

**Table 8:** In-orbit test results of the MAVIS under vibration excitation mode

Desired acceleration signal			Control strategy	Measurement		
Waveform	Frequency (Hz)	Amplitude (μg_0_)		Frequency (Hz)	Displacement (mm)	Acceleration (μg_0_)
Sine wave	0.01	0.4	SDC (2 mm)	0.00995	1.894	0.4091
Sine wave	0.1	40	SDC (2 mm)	0.1009	1.254	37.09
Sine wave	1.0	300	SDC (2 mm)	1.0	0.1354	299.9
Mixed sine waves	0.01	0.2	SDC (2 mm)	0.009929	0.8115	0.1803
	0.1	20	SDC (2 mm)	0.1	0.6426	19.26
Sine wave	10	4500	TIADC (60,000 μg_0_)	9.624	—	4355
Triangular wave	1.0	200	TIADC (1000 μg_0_)	0.999	—	219
Triangular wave	10	700	TIADC (6000 μg_0_)	9.61	—	702.5
Mixed sine waves	1.0	70	TIADC (1000 μg_0_)	0.9999	—	65.84
	10	6000	TIADC (60,000 μg_0_)	9.999	—	6253

### Conclusion

The MAVIS is a component of the FRP research platform designed to isolate the payload of the FPR from disturbances arising from the space station itself in the microgravity operating mode, while providing an environment with controllable vibrational acceleration signals of specific amplitudes in the frequency range of 0.01–10 Hz in the vibration excitation operating mode. The design and in-orbit test results of the MAVIS were presented. The primary results and analyses can be summarized as follows.

1. The controller combing feedforward and feedback design based on the applied dynamics models and the method employed for calculating the PID controller parameters using the closed-loop transfer function are effective.
2. Relatively low-frequency acceleration vibration signals can be indirectly achieved with the SDC strategy, while relatively high-frequency vibration signals can be achieved directly with the TIADC strategy.
3. The umbilicals applied between the floater and payload are major factors affecting the vibration isolation performance of the MAVIS. The pretensioning force and torque of the umbilicals are approximately equal to the output force and torque from the controller under steady-state in-orbit closed-loop control. The stiffness of the umbilicals can be estimated by comparing the microgravity accelerations of the stator and floater under the state of free levitation.
4. The translational and rotational motions of the floater are coupled in the control process, and their interactions must be considered in the design of the control system.
5. The large stiffness of the umbilicals enables the use of a negative coefficient for the proportional term of the PID controller to decrease the control bandwidth of the MAVIS effectively and improve the microgravity acceleration level of the floater.
6. The MAVIS achieved a microgravity level of 1–30 μg_0_ in the frequency range of 0.01–125 Hz and attenuated the magnitude of disturbances at frequencies greater than 2 Hz by 10-fold in the microgravity operating mode. In the vibration excitation operating mode, the MAVIS generated a minimum vibration acceleration of 0.4091 μg_0_ at a frequency of 0.00995 Hz and a maximum vibration acceleration of 6253 μg_0_ at a frequency of 9.999 Hz.

Accordingly, these findings confirm that the MAVIS provides a highly stable environment for conducting microgravity experiments, and promotes the development of microgravity fluid physics.

## Supplementary information

Reporting Summary

## Data Availability

The datasets analyzed during the current study available from the corresponding author on reasonable request.

## Code availability

The underlying code for this study is not publicly available but may be made available to qualified researchers on reasonable request from the corresponding author.
